# The combined effect of gaze stability and balance exercises using telerehabilitation in individuals with vestibular disorders during the COVID-19 pandemic: A pilot study

**DOI:** 10.1371/journal.pone.0282189

**Published:** 2023-05-05

**Authors:** Nada Aldawsary, Maha Almarwani

**Affiliations:** 1 Department of Health Rehabilitation Sciences, College of Applied Medical Sciences, King Saud University, Riyadh, Saudi Arabia; 2 Department of Medical Rehabilitation, Physical Therapy Department, Ministry of Health, Riyadh, Saudi Arabia; Prince Sattam Bin Abdulaziz University, College of Applied Medical Sciences, SAUDI ARABIA

## Abstract

**Background:**

Vestibular rehabilitation is recognized as the most effective intervention to relieve symptoms of dizziness and imbalance related to vestibular disorders.

**Objective:**

This study aimed to examine the combined effect of gaze stability and balance exercises using telerehabilitation in individuals with vestibular disorders during the COVID-19 pandemic.

**Methods:**

This pilot study was a quasi-experimental, single-group design pre- to post-telerehabilitation intervention. Individuals with vestibular disorders between the ages of 25–60 participated in this study (n = 10). Participants underwent four weeks of combined gaze stability and balance exercises using telerehabilitation at their homes. The Arabic version of the Activities-Specific Balance Confidence scale (A-ABC), Berg Balance Scale (BBS), and the Arabic version of the Dizziness Handicap Inventory (A-DHI) were assessed pre- and post-vestibular telerehabilitation. Wilcoxon signed rank test was used to examine the magnitude of difference pre- and post-intervention scores of outcome measures. The effect size (r) for the Wilcoxon signed rank was calculated.

**Results:**

After four weeks of vestibular telerehabilitation, there was an improvement in BBS and A-DHI outcome measures (*p* < .001), with moderate effect size for both scales (r = 0.6). However, A-ABC showed no significant improvement among participants.

**Conclusion:**

This pilot study found that the combined effect of gaze stability and balance exercises using telerehabilitation appear to be effective in improving balance and activities of daily living in individuals with vestibular disorders.

## Introduction

Vestibular disorders are common and have a significant impact on overall health. Individuals with vestibular disorders complain of sudden, prolonged, and severe dizziness, horizontal rotatory nystagmus, nausea, and balance deficits, which increase the risk of falling and reduce the quality of life [1–5]. Vestibular rehabilitation is widely recognized as the most effective intervention to relieve symptoms of dizziness and imbalance associated with vestibular disorders and improve activities of daily living [6–15].

The Neurology Section of the American Physical Therapy Association recommended practicing 20–30 min of gaze stability 4–5 times a day in combination with 20 min of balance exercise for four weeks for individuals with vestibular disorders [9]. Adding balance exercises to gaze stability exercises aims to substitute for missing vestibular function using visual and somatosensory cues [16]. In 2020, the COVID-19 pandemic altered health care worldwide, and the World Health Organization approved postponing non-urgent treatment to guarantee a safe rehabilitation service [17–19]. It was challenging to deliver physical therapy services to patients; therefore, there was an urgent need to provide physical therapy using telerehabilitation.

Telerehabilitation has the potential to improve quality of life and can be as effective as conventional care [19–22]. It can play an important role in mitigating the spread of COVID-19 and supporting public health precautions. Global health organizations, including the Saudi Ministry of Health, have suggested resources to implement telerehabilitation [19, 23–25]. Telehealth applications were used by more than two million patients with positive attitudes in Saudi Arabia [25–27].

A recent study in the USA reported that 86% of physical therapists agreed that telehealth could be used effectively for individuals with vestibular disorders, with similar outcomes as in-person clinic care [28]. In addition, patients from vestibular outpatient clinics were reported to be satisfied after practicing physical therapy services using telerehabilitation [29]. Although there is supportive evidence for vestibular telerehabilitation’s effectiveness [28, 29], no study has assessed the effect of vestibular telerehabilitation in Arabic-speaking individuals with vestibular disorders. Therefore, this study aimed to examine the combined effect of gaze stability and balance exercises using telerehabilitation in individuals with vestibular disorders during the COVID-19 pandemic. We hypothesized that the combination of gaze stability and balance exercises using telerehabilitation would positively affect balance and activities of daily living among individuals with vestibular disorders.

## Materials and methods

### Study design

This pilot study was a quasi-experimental, single-group design pre- to post-telerehabilitation intervention. The authors confirm that all ongoing and related trials for this intervention was registered with ClinicalTrials.gov (NCT04842474).

### Participants

During COVID-19 physical therapy services were not allowed in the clinic, therefore the recruitment rate was limited and the decision to do pilot study was made. Individuals with vestibular disorders between 25–60 years of age were recruited and assessed between May 1, 2021 and July 1, 2021 for participation in this study. The study sample was recruited from various ear, nose, and throat (ENT) clinics in Riyadh, Saudi Arabia. Individuals were included if they were cognitively intact and capable of completing the questionnaire/assessments, clinically diagnosed with vestibular disorders, and had one or more of the following: (i) related burden symptoms that affected their daily activities; (ii) a history of dizziness/vertigo triggered by head or body movement; and (iii) independence or need for reasonable assistance. Individuals were excluded if they (i) had a history of neurological deficit; (ii) had a previous surgery that could affect balance or cause dizziness; (iii) had orthopedic problems that prohibited the performance of the exercises; (iv) reported spontaneous episodes of dizziness, which were not worsened by movements; and (v) were unable to follow instructions or completely dependent on the assistive device during mobility.

Eligible individuals were screened using video calls. This study was approved by the College of Medicine Institutional Review Board at King Saud University (IRB #E-20-5496), and all participants provided informed consent prior to participation.

### Outcome measures

#### The Arabic version of the Activities-Specific Balance Confidence Scale (A-ABC)

It measures a person’s self-perceived level of confidence in their ability to maintain the balance required for daily functional activities. Confidence in balance rates on a scale from 0–100%, with higher scores indicating a greater confidence in maintaining balance [30, 31]. A previous study showed that the A-ABC is reliable and valid and can be used in Arabic-speaking individuals with vestibular disorders [32].

#### Berg Balance Scale (BBS)

It evaluates the functional balance using 14 tasks: sitting to standing, standing unsupported, sitting unsupported, standing to sitting, transfers, standing with eyes closed, standing with feet together, reaching forward with an outstretched arm, retrieving object from the floor, turning to look behind, turning 360 degrees, placing the foot alternately on a step, standing with one foot in front, and standing on one foot. The maximum score was 56 points, representing normal balance. Each item was scored from 0 (unable to perform) to 4 (normal performance). Previous studies have shown that the BBS has good reliability and validity in individuals with vestibular disorders [33, 34].

#### The Arabic version of the Dizziness Handicap Inventory (A-DHI)

It assesses the self-perception of the incapacitating effects provoked by dizziness. The scale contains 25 items, and a total score (0–100 points) is obtained by summing ordinal scale responses, with higher scores indicating a more severe handicap. The scale was developed to capture various subdomains of self-perceived handicap and comprises seven physical, nine functional, and nine emotional questions [31, 35]. A previous study has shown that the A-DHI has good reliability and validity in Arabic-speaking individuals with vestibular disorders [36].

### Intervention

The combination of gaze stability and balance exercise was performed as scheduled in the treatment plan [9, 37] (Table 1). The participants were instructed to perform the exercises twice on alternate days for four weeks. Each session lasted for 45–60 minutes. The rest interval was five minutes between the two forms of exercises.

**Table 1:** Intervention plan during four weeks of training including gaze stability exercises (GSEs) and balance exercises (BE).

Week	Intervention		Frequency	Duration
	GSEs (Sitting)	BE (Standing)		
1& 2	**Eyeball;** moving the eyes to a different direction slowly while they were closed to the left and right, up and down, rotation movements **Saccadic;** moving the eyes as quickly as possible between the stationary points with a fixed head **Pursuit;** tracking slowly moving target with the eyes without moving head **Vergence eye movement;** tracking the moving target from 5 cm close to eye level to as far as possible, both backward and forward. **Vestibulo-ocular reflex;** keeping the eyes on the fixed point while moving the head from left to right.	Standing on a firm surface with feet apart, heel stand, toe stand, marching on a firm surface, semi tandem stand, walking forward and backward with a normal support base **(with eyes open)**	Twice/alternatively day.	Each GSEs lasted 30 seconds. BE 20 lasted minutes. Rest between GSEs & BE was 5 minutes.
3& 4	Same as week 1 & 2.	Same as week 1 & 2 **(with eyes closed).**	Same as week 1 & 2.	Same as week 1 & 2.

The gaze stability exercises (GSEs) included five phases: eyeball movement, saccadic, pursuit, vergence eye movements, and vestibulo-ocular reflex (VOR) exercise. The eyeball movement exercise moved the eyes in a different direction slowly while they were closed to the left and right, up, and down, and rotated. The saccadic eye movement exercise moved the eyes as quickly as possible between stationary points with a fixed head. Pursuit eye movement involved tracking slow-moving targets with the eyes without moving the head. The vergence eye movement exercise involved tracking the moving target from 5 cm close to the eye level to as far as possible, both backward and forward. The VOR exercise involved keeping the eyes at a fixed point while moving the head from left to right. GSEs were performed while the patients were seated, and each exercise lasted 30 seconds [9, 37].

Static and dynamic balance exercises were recommended to improve postural stability, with or without closing the eyes. In the first two weeks, the participants performed balance exercises with their eyes open, and in the next couple of weeks, they performed the same exercises with their eyes closed. It involves standing on a firm surface with feet apart, heel stand, toe stand, marching on a firm surface, semi-tandem stand, and walking forward and backward with a normal support base [9, 37].

### Procedure

After the participant agreed and signed the consent form virtually, the participants’ demographic and medical data were obtained. After completing the baseline assessment, participants in this study were instructed to follow videos demonstrating the combination of gaze stability and balance exercises with audio descriptions in Arabic. In addition, all participants were telemonitored weekly for four weeks at home by physical therapists using video calls. All outcome measures were measured at baseline and after four weeks of vestibular telerehabilitation intervention.

### Statistical analysis

All statistical analyses were performed with IBM SPSS Statistics version 25 (Armonk, NY, USA). Descriptive statistics were used to summarize the characteristics of participants and the outcome measures for the study. Frequency (percentage) was used to describe categorical variables. Mean (standard deviation) or median (interquartile range [Q25–Q75]) were used to describe continuous variables. The non-parametric Wilcoxon signed-ranks test was calculated to examine the difference in the A-ABC, BBS, and A-DHI scores pre-to post-vestibular telerehabilitation intervention. The effect size (r) for the Wilcoxon signed rank was calculated using the following formula: r = *Z*/√*N* [38], where N is the total number of the samples. The effect size (r) was interpreted following guidelines suggested by Cohen [38, 39]. Statistical significance was set a priori at two-sided *p* < .05.

## Results

**Fig 1:**
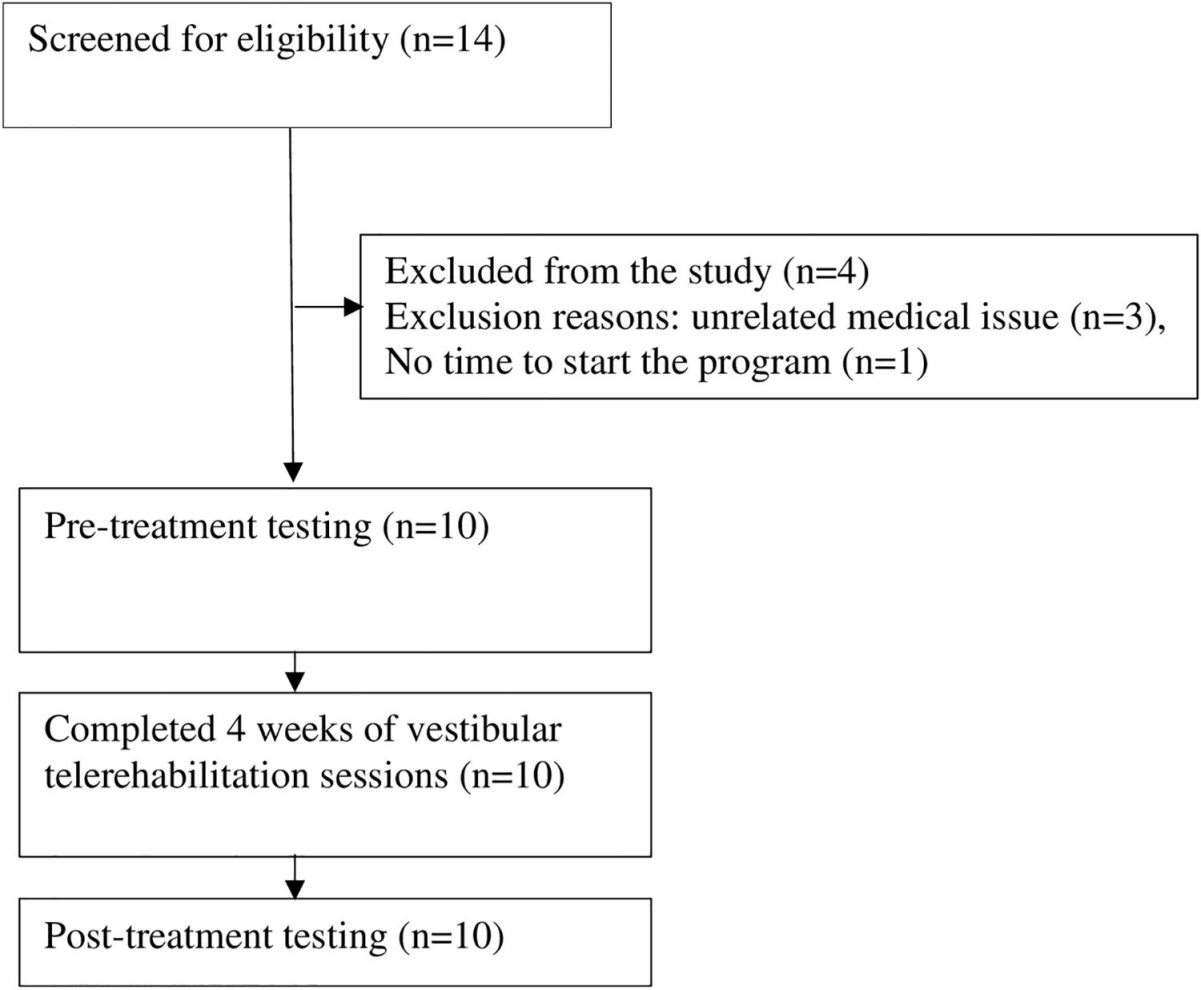
The participants flow chart.

A total of 14 individuals with vestibular disorders were screened for participation in this study. Based on our exclusion criteria, three were excluded, and one dropped out. Ten participants conducted the study program and completed the designed intervention. The participant flow chart and reasons for dropout are shown in (Fig 1).

Five males and five females were enrolled in the study with a mean age of 40.90 with a standard deviation of 11.24 years. Three participants were diagnosed with vertigo due to vestibular neuritis (*n* = 3), five with dizziness related to vestibular dysfunction (*n* = 5), and two with migraine vestibulopathy (*n* = 2) (Table 2).

**Table 2:** Characteristics of participants (n = 10).

Variables	N (%)
***Gender***	
Male	5 (50)
Female	5 (50)
***Age (y)*** *	40.90 ±11.24
***Diagnosis***	
Neuritis Migraine related vestibulopathy Dizziness related to vestibular disorders	3 (30) 2 (20) 5 (50)

Note: * age presented as mean and standard deviation (M±SD).

The descriptive statistics of the study variables for the outcome measure (A-ABC, BBS, A-DHI) pre and post interventions are presented in (Table 3). For participants with vestibular disorders who were treated with vestibular telerehabilitation, there was a significant improvement in BBS and A-DHI outcome measures scores after four weeks of intervention compared with initial assessment (*p* < .001). The effect size (*r*) of improvements was moderate for both BBS and A-DHI (*r* = 0.6). However, A-ABC scale demonstrated a non-significant improvement within participants post completing exercises (*p =* .262).

**Table 3:** Outcome measures pre- to post vestibular telerehabilitation intervention (n = 10).

Variables	Pre^a^	Post^a^	Difference between pre and post^b^
**A-ABC**	11.5 (8.1–13.1)	10.8 (8.6–14.4)	Z = -1.122, *p =* 0.262, r = 0.3
**BBS**	46.5 (33.5–51.0)	54.0 (47.5–56.0)	Z = -2.810, *p* < .001, r = 0.6
**A-DHI (Total score)**	56.0 (29.0–77.0)	22.0 (12.0–58.5)	Z = -2.668, *p* < .001, r = 0.6
**A-DHI (Physical domain)**	6.0 (3.0–16.0)	18.0 (9.0–22.5)	Z = -2.527, *p* < .001, r = 0.6
**A-DHI (Emotional domain)**	6.0 (1.5–8.0)	17.0 (5.5–30.5)	Z = -2.316, *p* < .001, r = 0.5
**A-DHI (Functional domain)**	10.0 (6.0–24.0)	14.0 (10.5–27.0)	Z = -1.407, *p* = 0.159, r = 0.3

Abbreviations: *r*, effect size for the Wilcoxon signed-rank test

aMedian (Q25-Q75).

bWilcoxon signed rank test.

## Discussion

We examined the combined effect of gaze stability and balance exercises using telerehabilitation in individuals with vestibular disorders during the COVID-19 pandemic. This study is the first to examine vestibular telerehabilitation in Arabic-speaking individuals with vestibular disorders. In our study, vestibular telerehabilitation showed general improvement in vestibular symptoms and related disabilities among individuals with vestibular disorders. We observed a significant improvement in the balance (Berg Balance Scale [BBS]), disability (the Arabic version of the Dizziness Handicap Inventory [A-DHI]) after four weeks of vestibular telerehabilitation. The results of this study were consistent with previous studies by Geraghty et al. (2017) and van Vugt et al. (2019) who concluded that Internet-based vestibular rehabilitation improved dizziness, vestibular symptoms, and related disabilities [6, 40].

Balance was objectively assessed through the BBS using a video call, and the outcomes were significantly improved with a moderate effect size (r = 0.6) in balance scores. These improvements could be attributed to vestibulo-ocular reflex adaptation through the performance of specific vestibular exercises and could be related to the enhancement of neuroplasticity with repetitive exercises for gaze stability and balance [41–43].

Based on the A-DHI scores, most participants in our study had a high level of dizziness-induced disability that affected their performance in activities of daily living, and all of them showed improvement after four weeks of intervention in the physical and emotional subscales with moderate effect size (r = 0.5–0.6). Similarly, Giray et al. (2009) reported an improvement in the emotional, physical, and functional subscales of the A-DHI, with increased quality of life and independence in individuals with chronic vestibular disorders after practicing four weeks of vestibular rehabilitation compared to the control group who did not receive any treatment [16].

However, in our study the functional subscale did not show an improvement among our participants may be because they had mild functional disabilities according to A-DHI measures.

The self-perception confidence level measured by the Arabic version of the activities-specific balance confidence scale (A-ABC) did not improve significantly after four weeks of vestibular telerehabilitation. This could be related to the short study duration compared to the previous studies that used internet-based vestibular rehabilitation by measuring the vertigo symptom scale-short form, with subjective improvement in outcomes in the dizziness score after six weeks of intervention [6, 40].

Currently, telerehabilitation represents the easiest and safest way to communicate with patients. It has continued after the pandemic in different medical services worldwide [28, 44–47]. Vestibular telerehabilitation could provide a reasonable alternative to in-person visits during the COVID-19 pandemic and beyond.

### Study limitations

Our study had a few limitations. First, the sample size was small, which affected the study’s generalizability. Second, it is limited by the difficulties in collecting target samples owing to the time of COVID-19. Third, objective balance assessment is more challenging for telerehabilitation users than in-person evaluations. Finally, the method of collecting the participants recruited from one region may have a potential selection bias.

Future randomized controlled studies should examine the long-term effects of gaze stability and balance exercises using telerehabilitation on larger sample sizes and compare vestibular telerehabilitation with conventional vestibular rehabilitation.

## Conclusions

This pilot study found that the combined effect of gaze stability and balance exercise using telerehabilitation is beneficial with positive outcomes, such as improved balance and independence in performing activities of daily living in patients with vestibular disorders.

## Supporting information

S1 Checklist (DOCX)

S1 Protocol (DOCX)
