# Supplementary material for: The combined effect of gaze stability and balance exercises using telerehabilitation in individuals with vestibular disorders during the COVID-19 pandemic: A pilot study
Source: PLoS One. 2023 May 5;18(5):e0282189. doi: 10.1371/journal.pone.0282189 (PMC10162509; doi:10.1371/journal.pone.0282189)
Supplement: S1 Protocol — (DOCX) [file pone.0282189.s002.docx]

**Effectiveness of Gaze Stability Exercises on Balance and Activities of Daily Living among Patients Suffering Vertigo with Vestibular Neuritis in Saudi Arabia: A pilot clinical trial, telerehabilitation study**

Nada Aldawsary, BS, PT^a,b^, Maha Almarwani, PhD, PT^a^

^a^Department of Rehabilitation Sciences, College of Applied Medical Sciences, King Saud University, Riyadh, Saudi Arabia

^b^Department of Medical Rehabilitation, Physical Therapy Department, Ministry of health, Saudi Arabia

We confirm that there are no known conflicts of interest associated with this publication.

# **ABSTRACT**

# **Background:**

Gaze stability exercise is a medical procedure for persons with unilateral vestibular disturbances such as vestibular neuritis or persons who have had tumors of their 8^th^ nerve. They are a crucial part of the vestibular dysfunction rehabilitation protocols in health centers. These activities which involve turning one's eyes at different angles while having their eyes focused on an optotype are aimed at helping improve the vestibular ocular reflex (VOR), visual acuity during head movements and also reducing vertigo and dizziness.

# **Study aim**:

This review will be establishing the effectiveness of applying gaze stability with balance exercises procedure among participants who are suffering vertigo due to vestibular neuritis.

**Methods:**

Twenty volunteers between the age of 40-59 years old, diagnosed, and confirmed to be suffering from vestibular neuritis and vertigo will be used in this study. Gaze stability exercises will be performed while patients are in a seated position. Each exercise will last for 30 seconds and be done in phases that included; eyeball movement, saccadic eye movement, pursuit eye movement, vergence eye movement, and vestibular-ocular reflex exercise. Balance exercises will be performed in a standing position including both static and dynamic training with or without closing eyes. The following outcome measures for each participant will be assessed pre-and post-treatment after completing four weeks of intervention. They include; Arabic version of Activities-Specific Balance Confidence Scale (A-ABC scale), Berg Balance Scale (BBS), and Katz Index of Independence in Activities of Daily Living (Katz ADL). The findings will then be subjected to statistical methods and data analysis using the SPSS toolkit.

**Key words:** vestibular neuritis, gaze stability, balance exercise

# **1 Background Information**

Balance and stability are important components for daily activities. Individuals need appropriate posture and coordination of various body parts for them to perform basic movements needed when doing various activities. The body requires a strong balance to perform day-to-day activities such as walking up and downstairs, bending, as well as lifting things from the ground. Lack of this balance, which can occur because of ear infections, accidents, and aging, generally can result in decreased quality of life. Nichols-Larsen et al., (2016) mentioned that vestibular neuritis is the second most common cause of vertigo and patients are complained of balance disturbance after head movements. Most healthcare facilities as well as health fitness centers use exercises to improve balance and stability in patients with balance problems. Examples of such balance exercises include gaze stability exercises. This literature reviews the effectiveness of gaze stability with balance exercises in enhancing balance, activities of daily living and reducing the risk of falling among patients suffering from vertigo due to vestibular neuritis in Riyadh.

## **1.1 Overview of Gaze Stability Exercises**

According to Gaikward (2016), Gaze stability exercises are part of vestibular rehabilitation therapy exercises. The exercises have become part of healthcare plans for patients with vestibular dysfunction (Gaikward, 2016). The exercises aim at improving vestibular ocular reflex (VOR), visual acuity during head movements, as well as reducing vertigo and dizziness (Gaikward, 2016). The therapy also improves postural stability, hence improving the quality of life among those affected (Gaikward, 2016). This, in turn, helps the patients achieve their activities of daily living (Clendaniel, 2010). Gaikward also mentions that the therapy works by promoting and stimulating vestibular adaptation and substitution to various stimuli provided during the exercises. Horizontal and vertical head movements with a fixed vision on a certain target induce retinal slip, which in turn increases the gain of vestibular response (Gaikward, 2016). However, achieving sustained vestibular response requires repeated exercises. Thus, most gaze stability exercises are prescribed for a long duration to achieve this response. Commonly used prescription is about four to five exercises in a day (Gaikward, 2016). The total exercises should last for an average of thirty minutes.

## **1.2 Effectiveness of Gaze Stability Exercises**

There are several studies and researches on vestibular rehabilitation therapy. A systematic analysis of most of these studies shows gaze stability exercises to be effective in achieving balance and postural stability (Kundakci et al., 2018, p.8). A recent study by Roh and Lee (2019) found a positive correlation between vestibular rehabilitation therapy and the improvement of balance and stability. According to their results, dynamic postural stability among patients with balance problems significantly improved after providing gaze stability exercises twice per week for four weeks. (Roh and Lee, 2019). The researchers used various forms of gaze stability exercises, including, eyeball, pursuit, saccadic, and vergence eye movements as well as vestibular ocular reflex exercise (Roh and Lee, 2019). Thus, these exercises proved effective in reducing vertigo, dizziness, and achieving balance among those affected.

Vestibular rehabilitation therapy also improves the overall health status of affected individuals as well as increasing the quality of their life. Tsukamoto et al., (2015) found an improvement in the quality of life among twenty patients diagnosed with vestibular disease. The intensity of dizziness also improved among these patients, with postural stability and balance improving in more than eight percent of the patients (Tsukamoto et al., 2015). Thus, the negative impacts of various vestibular diseases, including vestibular neuritis, can be minimized by prescribing well-coordinated and supervised gaze stability exercises.

Khanna and Singh (2014) saw related findings in another study. However, according to their results, improvement in postural stability and balance requires repeated exposure to interventions for a significant duration. The duo provided gaze stability exercises for two consecutive days per week for a duration of six weeks (Khanna and Singh, 2014). The patients conducted the exercises three times per day with each session lasting for forty to sixty minutes (Khanna and Singh, 2014). Moreover, vestibular rehabilitation therapy to be more effective if two or more types of exercises can be combined during the interventions. Those who were receiving balance training in addition to gaze stability exercises had a significant improvement in their balance and stability compared to those receiving either of the two therapies alone (Khanna and Singh, 2014).

Although vestibular rehabilitation therapy is generally effective, some studies have shown a negative side of them. According to Luth et al., (2019), the therapy is only effective for a short term, hence making them less effective than conservative as well as medical therapy. According to the researchers, vestibular rehabilitation therapy works only for a maximum of three weeks (Luth et al., 2019). The findings in this study can limit the use of vestibular rehabilitation therapy, including gaze stability exercises in those with long-term disabilities. However, combining various forms of exercises may remove this weakness, hence making vestibular rehabilitation therapy an interesting area for future researches.

## **1.3 Vestibular Rehabilitation in Saudi Arabia**

There is a consensus among scholars over a recently published study that presents concise and well-researched investigations on vestibular rehabilitation therapy. The study discusses vestibular rehabilitation therapy and its positive effects on vestibular dysfunction patients. Furthermore, this study stresses Vestibular rehabilitation as an effective form of therapy in alleviating the symptoms of various vestibular diseases such as vestibular neuritis. The therapeutic exercises are beneficial in attaining post stability and improving balance in patients with balance issues (Macias JD, Massingale S and Gerkin RD, 2005).

However, studying the application of the combination of gaze stability and balance exercises to improve daily living activities in patients suffering from vertigo with vestibular neuritis in Saudi Arabia poses challenges since there is a lack of published data in the department of Saudi Arabia. There are a few qualified specialists in Saudi Arabia practicing VRT besides it is not commonly used in local physiotherapy departments. The pandemic of Covid 19- has made it difficult for patients to receive physical therapy services that enhance telerehabilitation role. Therefore, this research designs need to look for ways to overcome those limitations by integrating other forms of exercises such as gaze stability and balance exercises with vestibular neuritis patients through telerehabilitation.

According to the existing literature, vestibular rehabilitation therapy is effective in alleviating the symptoms of various vestibular diseases such as vestibular neuritis. The exercises are useful in achieving postural stability and improving balance among patients with balance issues. However, the studies have some limitations since most of the researchers did not follow up on their participants for long enough to ensure they had long-term remissions. Thus, there is an opportunity to conduct more studies to ascertain the effectiveness of gaze stability exercises including the quality of patients' life among Saudi population.

## **1.4 Objectives**

This literature aims to assess whether conducting gaze stability with balance exercises has an improvement influence on balance and reducing falling incidence. Moreover, to study the impact of applying combination of gaze stability and balance exercises to improve activities of daily living in patients suffering from vertigo with vestibular neuritis in Saudi Arabia.

# **2 METHODOLOGY**

## **2.1 Study design**

This research is experimental with Pre-post cohort design, Pilot clinical trial, by using telerehabilitation.

## **2.2 Participants**

Middle-aged adults of 40-59 years old suffering vertigo due to vestibular neuritis. Through the following criteria, all participants will be eligible to attend the study.

### **2.2.1 Inclusion/exclusion criteria**

A convenience volunteers of middle-aged adults of 40-59 years old are diagnosed with vestibular neuritis and vertigo. Participants should not be either with a previous history of neurological diseases or brain surgery affecting equilibrium. Both male and female are cognitively intact. Subjects with medical issues, e.g., hypertension and diabetes, are undertaking medication regularly. Independent participants or those requiring reasonable assistance, but with balance disturbance in daily living activities and transferring, having Berg Balance Scale between 21-56 are included in the study.

However, subjects with the inability to follow instructions or completely dependent on the assistive device during mobility have been eliminated from the study. Participants with chronic dizziness diagnosed specifically with benign paroxysmal positional vertigo (BPPV), migraine-associated vertigo, or Meniere's disease are excluded. The research will be applied locally in Saudi Arabia, and all participants will sign a consent form approved by the Ethics Committee of King Saud University.

## **2.3 Sample size**

Twenty convenient volunteers of middle-aged adults of 40-59 years old (n=20) are diagnose with vestibular neuritis and vertigo. The sample size is the same as previous studies (Roh and Lee, 2019).

## **2.4 Intervention**

The gaze stability exercises (GSEs) and balance exercises (BE) are described by Roh& Lee (2019) and Khanna& Singh (2014), respectively. This research is reconstructed by combining two forms of exercises (GSEs)& (BE). Participants with vestibular neuritis and vertigo will be practicing a combination of gaze stability exercise (GSEs) and balance exercises (BE).

Gaze stability exercises are included in five phases: eyeball movement, saccadic eye movement, pursuit eye movement, vengeance eye movement, and vestibular-ocular reflex exercise (figure 1) (Appendix 2) (Roh and Lee, 2019). The eyeball exercise moves the eyes to a different direction slowly while they are closed to the left and right, up and down, rotation movements (Roh and Lee, 2019). The saccadic eye movement exercise moves the eyes as quickly as possible between the stationary points with a fixed head (Roh and Lee, 2019). The pursuit eye movement exercise by tracking slowly moving target with the eyes without moving head (Roh and Lee, 2019). The vergence eye movement exercise includes tracking the moving target from 5 cm close to eye level to as far as possible, both backward and forward (Roh and Lee, 2019). The vestibular-ocular reflex exercise keeps the eyes on the fixed point while moving the head from left to right (Roh and Lee, 2019). Gaze stability exercises will be performed while patients are seated, and each exercise lasts for 30 seconds.

Balance exercises, both static and dynamic with or without closing eyes, are recommended to improve postural stability (Khanna and Singh, 2014). In the first two weeks, patients will perform balance exercises with opening eyes, and then in the next couple of weeks, participants will do the same exercises with closing eyes (table 1) (Appendix 1). It involves standing on a firm surface with feet apart, heel stand, toe stand, marching on a firm surface, semi tandem stand, walking forward and backward with a normal support base (Khanna and Singh, 2014).

This intervention of gaze stability with balance exercises will be given as scheduled in the treatment plan (table 1) (Appendix 1) (Roh and Lee, 2019). It will be applied twice each alternative day with ten repetitions for a total treatment period of four weeks. Each session will last for 45-60 minutes. Rest interval is between two groups of exercises for five minutes. Warm-up and cool down for five minutes is applicable before and after completing the treatment session. The patients will be followed up weekly through tele rehabilitation under the supervision of the researcher.

## **2.5 Outcome measures**

The following outcome measures for each participant will be evaluated before the commencement of treatment and at the end of four weeks of training. They include; Arabic version of Activities-Specific Balance Confidence Scale (A-ABC scale) to measure patient self-confidence for performing activities of daily living without losing balance. Permission has been granted from original author Dr. Alia Alghwiri by email to use the Arabic version of the ABC scale. It has proved its good reliability and validity and can be used with the Arab population's vestibular disorders (Alghwiri AA, Alghadir AH, Al-Momani MO and Whitney SL, 2015) (figure 2) (Appendix 3).

In addition, Berg Balance Scale (BBS) to evaluate static balance and fall risk in adult. It has shown good reliability and validity with vestibular patients too (Alghwiri AA, Alghadir AH, Al-Momani MO and Whitney SL, 2015) (figure 3) (Appendix 3).

Moreover, Katz Index of Independence in Activities of Daily Living (Katz ADL) to define problems in performing ADLs. It has demonstrated excellent validity and reliability to assess the ability to perform daily living activities independently in adult and older community-dwelling populations (Hopman-Rock M, van Hirtum, H, de Vreede P and Freiberger E, 2019) (figure 4) (Appendix 3).

## **2.6 Data analysis**

Data will be collected, tabulated, and analyzed using SPSS version 25. The descriptive data will be represented as mean (*M*) and standard deviation (*SD*). Paired *t*-test will be run to compare pre-intervention and post-intervention scores of A-ABC, BBS& Katz-ADL for each participant. Pearson’s r regression will be used to detect the linear association between variables. Results will be statistically significant at p <0.05.

## **2.7 Ethical approval**

Proposal will be submitted to the Institutional Review Board in the college of medicine at King Saud University for ethical approval prior to the start of the research.

1. **Time Schedule of the research team**

| **العام الأول** | | | | | | | | | **First Year** | | | | |
| --- | --- | --- | --- | --- | --- | --- | --- | --- | --- | --- | --- | --- | --- |
| **الشهور / Months** | | | | | | | | | | | | **المهمة / Task** | **S. No** |
| **12** | **11** | **10** | **9** | **8** | **7** | **6** | **5** | **4** | **3** | **2** | **1** |  |  |
|  |  |  |  |  |  |  |  |  |  |  |  | **Data collection** | **1** |
|  |  |  |  |  |  |  |  |  |  |  |  | **Intervention** | **2** |
|  |  |  |  |  |  |  |  |  |  |  |  | **Data analysis** | **3** |
|  |  |  |  |  |  |  |  |  |  |  |  | **Writing** | **4** |
|  |  |  |  |  |  |  |  |  |  |  |  |  | **5** |
|  |  |  |  |  |  |  |  |  |  |  |  |  | **6** |
|  |  |  |  |  |  |  |  |  |  |  |  |  | **7** |
|  |  |  |  |  |  |  |  |  |  |  |  |  | **8** |
|  |  |  |  |  |  |  |  |  |  |  |  |  | **9** |
|  |  |  |  |  |  |  |  |  |  |  |  |  | **10** |
|  |  |  |  |  |  |  |  |  |  |  |  |  | **11** |
|  |  |  |  |  |  |  |  |  |  |  |  |  | **12** |

**References:**

Alghwiri, A. A., Alghadir, A. H., Al-Momani, M. O., & Whitney, S. L., 2015. The activities- specific balance confidence scale and berg balance scale: Reliability and validity in Arabic-speaking vestibular patients. *Journal of Vestibular Research*, 25(5-6), 253-259.

Alyahya, D., 2015. Effect of vestibular adaptation exercises on chronic motion sensitivity.‏

Clendaniel, R. A., 2010. The effects of habituation and gaze-stability exercises in the treatment of unilateral vestibular hypofunction–preliminary results. *Journal of neurologic physical therapy:* JNPT, 34(2), 111.

‏

Gaikwad, S.B., 2016. Effect of Progressive Gaze Stability Exercises on Holistic Aspects of Chronic Motion Sensitivity.

Han BI, Song HS, Kim JS, 2011. Vestibular rehabilitation therapy: review of indications, mechanisms, and key exercises. *J Clin Neurol*.;7(4):184-96.

Herdman, S. J, 2013. Vestibular rehabilitation. *Current opinion in neurology*, *26*(1), 96-101.‏

Hopman-Rock, M., van Hirtum, H., de Vreede, P., & Freiberger, E, 2019. Activities of daily living in older community-dwelling persons: a systematic review of psychometric properties of instruments. Aging clinical and experimental research, 31(7), 917-925.

Khanna, T., and Singh, S., 2014. Effect of gaze stability exercises on balance in the elderly. *IOSR Journal of Dental and Medical Sciences (IOSR-JDMS)*, *1*, pp.41-48.

Kundakci, B., Sultana, A., Taylor, A.J., and Alshehri, M.A., 2018. The effectiveness of exercise-based vestibular rehabilitation in adult patients with chronic dizziness: A systematic review. *F1000Research*, *7*.

Luth, C., Bartell, D., Bish, M., Yudd, A., Palaima, M., and Cleland, J.A., 2019. The effectiveness of vestibular rehabilitation therapy vs conservative treatment on dizziness: a systematic review and meta-analysis. *Physical Therapy Reviews*, *24*(5), pp.229-238.

Macias, J. D., Massingale, S., & Gerkin, R. D., 2005. Efficacy of vestibular rehabilitation therapy in reducing falls. Otolaryngology—Head and Neck Surgery, 133(3), 323-325.

Mortazavi, H., Tabatabaeichehr, M., Taherpour, M., & Masoumi, M., 2018. Relationship between home safety and prevalence of falls and fear of falling among elderly people: a cross-sectional study. *Materia socio-medica*, 30(2), 103.‏

Nichols-Larsen, D. S., Kegelemeyer, D. A., Buford, J.A., et.al. (eds)., 2016. *Neurologic Rehabilitation: Neuroscience and Neuroplasticity in Physical Therapy Practice.* New York, McGraw-Hill Education.

Roh, M., and Lee, E., 2019. Effects of gaze stability exercises on cognitive function, dynamic postural ability, balance confidence, and subjective health status in old people with mild cognitive impairment. *Journal of Exercise Rehabilitation*, *15*(2), p.270.

Szturm T, Reimer KM, Hochman J., 2015. Home-based computer gaming in vestibular rehabilitation of gaze and balance impairment. *Games Health J*, 4(3):211-20.

Timothy C., 2019; Gaze exercises.

Tsukamoto, H.F., Costa, V.D.S.P., Silva Junior, R.A.D., Pelosi, G.G., Marchiori, L.L.D.M., Vaz, C.R.S. and Fernandes, K.B.P., 2015. Effectiveness of a vestibular rehabilitation protocol to improve the health-related quality of life and postural balance in patients with vertigo. *International archives of otorhinolaryngology*, *19*(3), pp.238-247.

**Appendix**

Table 1: Intervention plan during four weeks of training including gaze stability exercises (GSEs) and balance exercises (BE) with opening or closing eyes.

| Week | Intervention | Frequency |
| --- | --- | --- |
| 1^st^ & 2^nd^ | 1.GSEs including eyeball, saccadic, pursuit, vestibulo-ocular reflex& vergence eye movement exercises.  2.BE including static& dynamic balance exercises, with opening eyes | Twice/alternatively day, 10 repetition each exercise. |
| 3^rd^ & 4^th^ | 1. GSEs  2. BE, with closing eyes | Twice/alternatively day, 10 repetition each exercise. |


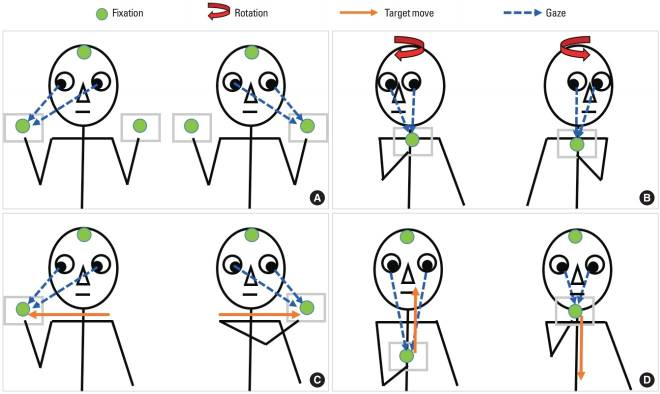


Figure 1: Four phases of Gaze Stability Exercises (GSEs) including (A) saccadic eye movement exercise, (B) pursuit eye movement exercise, (C) vestibulo-ocular reflex exercise, and (D) vergence eye movement exercise (Roh and Lee, 2019).

**
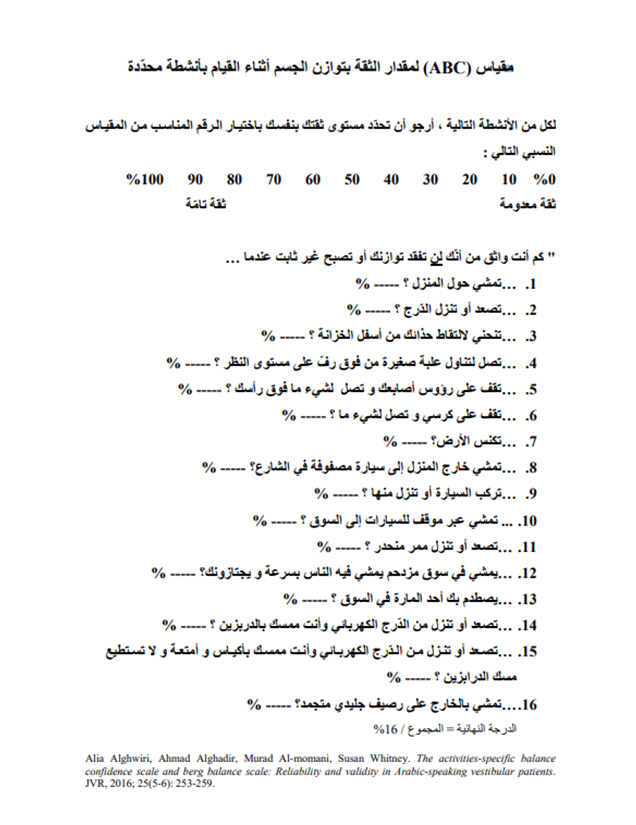
**

Figure 2: Arabic version of Activities-Specific Balance Confidence Scale (A-ABC scale) (Alghwiri AA, Alghadir AH, Al-Momani MO and Whitney SL, 2015).

**
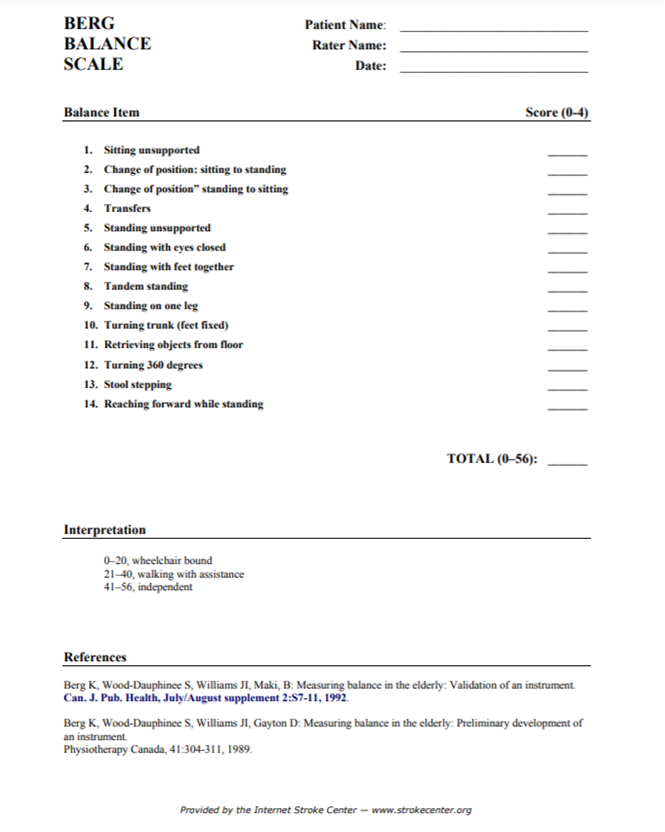
**

Figure 3: Berg Balance Scale (BBS) (Alghwiri AA, Alghadir AH, Al-Momani MO and Whitney SL, 2015).

**
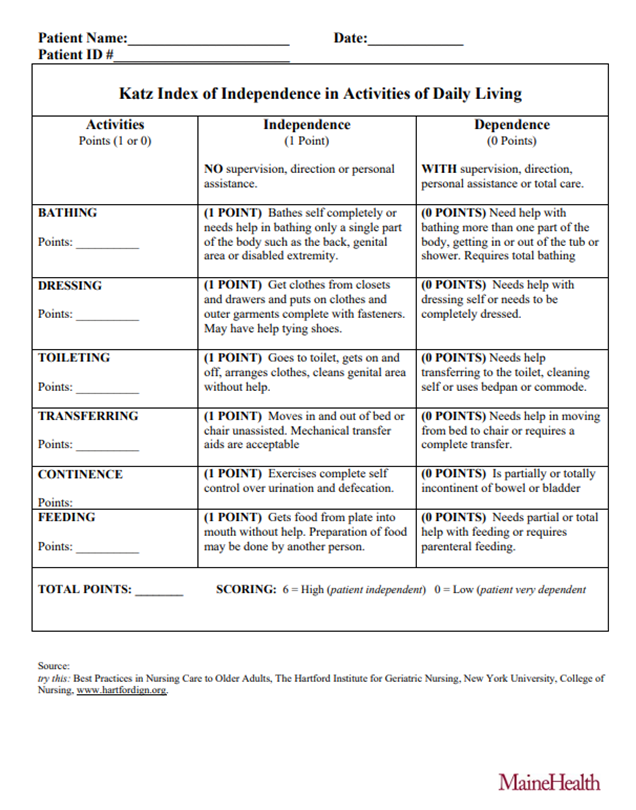
**

Figure 4: Katz index of independence activities of daily living (Katz ADL) (Hopman-Rock M, van Hirtum, H, de Vreede P and Freiberger E, 2019).

**
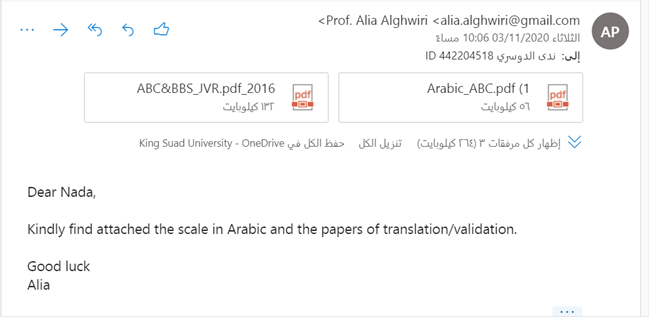
**

Figure 5: Permission is getting through an email to use A-ABC scale by author Professor Alia Alghwiri.
